# Aesthetic Depigmentation of Gingival Smoker's Melanosis Using Carbon Dioxide Lasers

**DOI:** 10.1155/2015/510589

**Published:** 2015-04-12

**Authors:** Luis Silva Monteiro, José Adriano Costa, Marco Infante da Câmara, Rui Albuquerque, Marco Martins, José Júlio Pacheco, Filomena Salazar, Fernando Figueira

**Affiliations:** ^1^Medicine and Oral Surgery Department and Institute of Research and Advanced Training in Health Sciences and Technologies (IINFACTS), Higher Institute of Health Sciences (ISCS-N), CESPU, 4585-116 Paredes, Portugal; ^2^Stomatology and Dental Medicine Department (CESPU), Centro Hospitalar de São João, Polo de Valongo, 4440-563 Valongo, Portugal; ^3^Oral Medicine Department, Birmingham Dental Hospital, School of Dentistry, University of Birmingham, Birmingham B4 6NN, UK

## Abstract

Melanic pigmentation results from melanin produced by the melanocytes present in the basal layer of the oral epithelium. One of the most common causes of oral pigmentation is smoker melanosis, a condition associated with the melanocyte stimulation caused by cigarette smoke. This paper aims to illustrate the use of a carbon dioxide laser in the removal of the gingival melanic pigmentation for aesthetic reasons in a 27-year-old female patient with history of a smoking habit. The carbon dioxide laser vaporisation was performed on the gingival mucosa with effective and quick results and without any complications or significant symptoms after the treatment. We conclude that a carbon dioxide laser could be a useful, effective, and safe instrument to treat the aesthetic complications caused by oral smoker melanosis.

## 1. Introduction

Gingivae are an important component of masticatory mucosa, contributing not only to the mastication process but also to anatomic and aesthetic characteristics of the individuals. The colour of the gums is determined by the thickness of epithelium, keratinisation degree, the presence and degree of melanin deposition, and the underlying connective tissue, including blood irrigation with presence of other pigments such as haemoglobin or oxyhemoglobin [1, 2]. The melanocytes are seen in the basal layer of the epithelium. They release melanin granules through the dendrite projections to the interior of the adjacent keratinocytes [3]. Melanin is a granular endogenous nonhaemoglobinic pigment that gives a brown or black colour (eumelanin) to the skin, mucosa, hair, and eye or sometimes a reddish colour (pheomelanin) [2]. Besides the colouration of the tissues the main function of this pigment is photoprotection, protecting the DNA from the UV rays [4].

The accumulation or increased deposition of melanin on oral mucosa can be physiological and called “racial pigmentation” or caused by several stimulants including trauma, infection, inflammation (recalcitrant lichen planus, lichenoid lesions, pemphigus, or pemphigoid), systemic disorders (Addison disease, Peutz-Jeghers disease, Laugier-Hunziker syndrome, or Albright disease), or drugs (clotrimazole, tetracycline, colchicine, and ketoconazole) [2, 5–7]. Smoking can also cause an excessive deposition of melanin in the oral epithelial layer of oral mucosa. Polycyclic amines such as nicotine and benzopyrenes, present in tobacco, can activate the melanocytes to produce melanin, perhaps as a protective adaptation of oral mucosa against tobacco agents [8]. Tobacco-associated melanin pigmentation (smoker melanosis) has been reported in 22% of smokers and is dose-dependent [9]. Women are more affected and the characteristic presentation is a diffuse black-brown macule that can involve mainly the gingiva, followed by buccal mucosa, lips, and hard palate [9]. Diagnosis is based on clinical characteristics and on smoking history in addition to the exclusion of physiological pigmentation, systemic causes such as Addison disease, hemochromatosis, and drug induced pigmentations [5]. Use of biopsy is an important method when malignancy is suspected or even to confirm clinical diagnosis [10]. Several techniques that have been developed aimed at the removal of the gingival melanic pigmentation for aesthetic reasons such as gingival surgical abrasions, scalpel gingivectomy, laser vaporization, cryosurgery, electrosurgery, chemical methods, and gingival grafts [11–18]. Lasers have been proposed as a useful method for the removal of the gingival melanic pigmentations. The advantages of lasers include haemostatic capacity, no need for sutures, and fewer postoperative complications such as pain, oedema, or infections [19–21]. The most used lasers are the carbon dioxide (CO_2_) laser (10,6 *μ*m) [15, 22, 23], Nd:YAG (1,064 *μ*m) [24], Er:YAG (2,94 *μ*m) [23, 25, 26], Argon (448 nm and 514 nm) [27], and high power diodes (808 nm, 810 nm, and 830 nm) [25, 28]. The aim of this paper is to:

- explain a case report, about a Portuguese female, who had CO_2_ laser treatment for the removal of gingival melanic pigmentation,
- carry out a literature review.

## 2. Clinical Case

**Figure 1:**
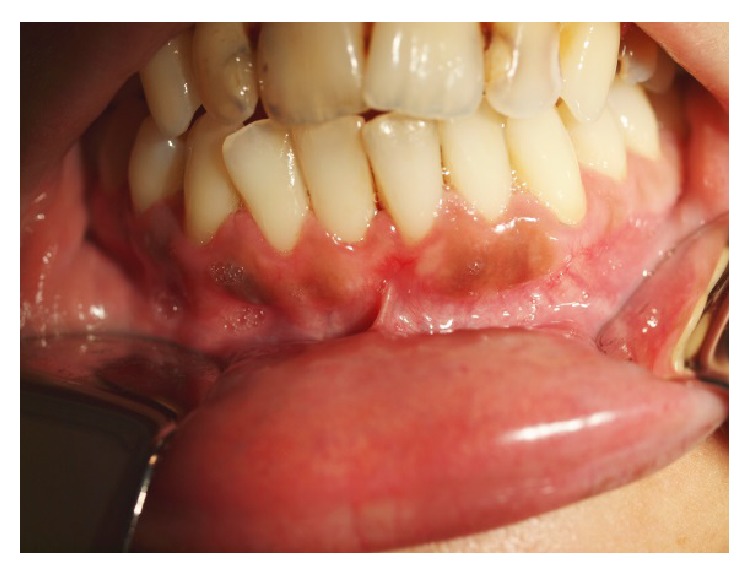
Initial clinical appearance with melanotic macules located on lower buccal gingiva.

**Figure 2:**
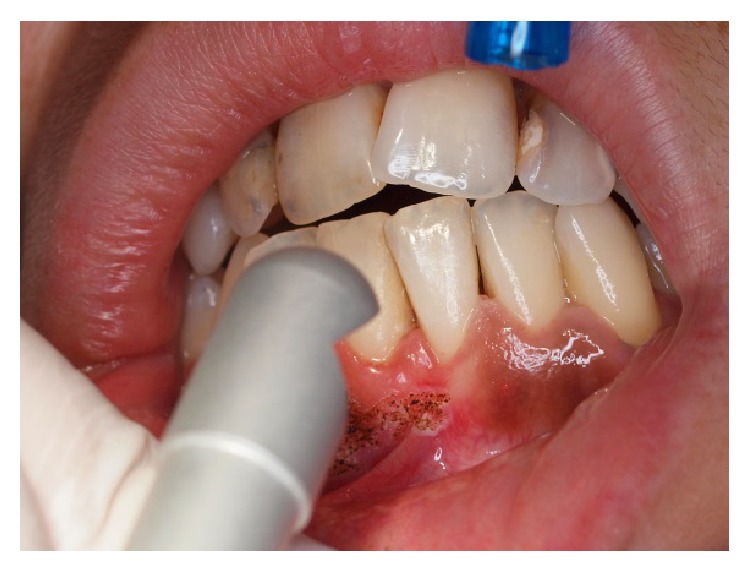
Vaporisation with CO_2_ laser of gingival macules.

**Figure 3:**
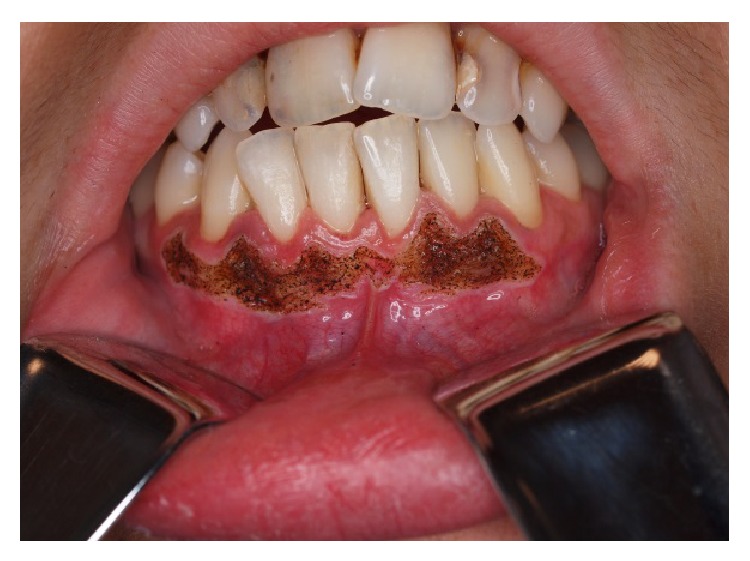
Clinical appearance after CO_2_ vaporisation procedure.

**Figure 4:**
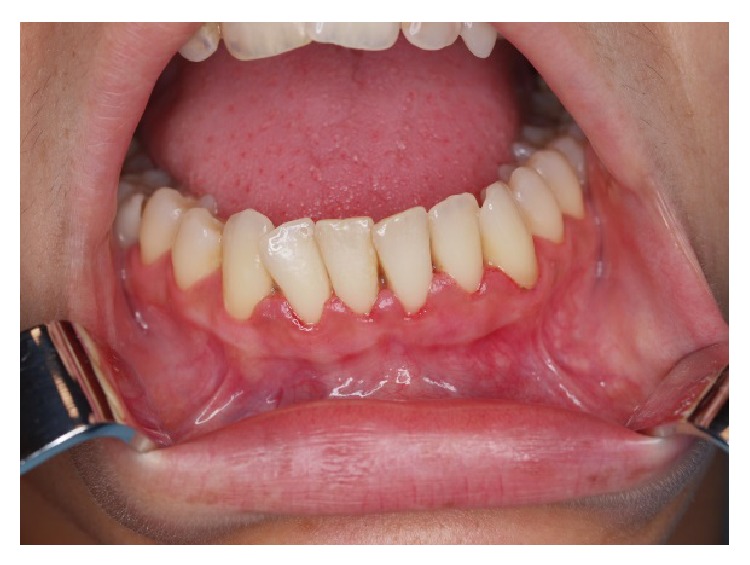
Clinical image of the lower anterior gingiva 3 weeks after CO_2_ vaporisation procedure without recurrence.

A 27-year-old Caucasian female was referred to the Stomatology and Dental Medicine Department (CESPU) of the Centro Hospitalar de São João, Pólo de Valongo, Oporto, Portugal. Her chief complaint was of a “dark-colour lower gum” and she made the request for rapid cosmetic therapy. She reported noticing darkening of the teeth and gums for over two years, mainly in the mandible. No symptoms and no significant personal or family history were present. The patient denied taking any medication or any other dark pigmentation in other locations. Her blood pressure was within the normal range of values. She had stopped smoking a year before the consultation, although she had smoked 30 cigarettes per day for more than 10 years. On oral examination two black-brown macules measuring 1 × 1 cm were detected in the vestibular attached lower gingiva (Figure 1). There was no lymphadenopathy or salivary gland abnormalities detected. Previous incisional biopsy to rule out any malignancy and blood investigations (complete full blood count and general biochemistry, including cortisol and ACTH serum levels) showed no abnormalities or potential causes. A diagnosis of smoker's melanosis was given based on the smoking history and the absence of any abnormalities seen in previous investigations. After explaining the benign character of the pigmentation, the patient requested the elimination of the pigmentation by aesthetic reasons due to professional work issues. A CO_2_ laser vaporization for gingival depigmentation was proposed. The vaporization of the pigmentation was performed under local anaesthesia (2% lidocaine with 1 : 100,000 epinephrine) with CO_2_ laser (10600 nm) (DEKA Smart US20D, Florence, Italy), with angulated mirror, focus spot of 1 mm, pulsed mode (50 Hz), 4.5 W of output power, power density of 573.25 W/cm^2^, and fluence of 11.46 J/cm^2^ (Figure 2). During this procedure, safety precautions were in place to protect the operator, assistant, and patient. With this laser procedure, the secondary-intention healing of the surgical wound was reached without any dressing (Figure 3). Paracetamol (1 g every 12 hours) was prescribed for two days to be taken only if she developed painful symptoms. After 3 weeks, wound healing was completed uneventfully without any melanin pigmentation (Figure 4). She did not report any postoperative pain, swelling, or other complications. After two years of follow-up, the patient had no symptoms or signs of gingival melanin pigmentation.

## 3. Discussion

The term “smoker's melanosis” was described by Hedin et al. in 1977 to characterise a benign limited melanin pigmentation occurring in the attached gingiva of tobacco smokers [29]. Through the stimulation, by polycyclic amines, smoking causes the activation of melanocytes to produce melanin [8]. These manifestations of pigmentation are considered normal and generally no treatment is recommended except for aesthetic purposes [2, 30]. Tobacco cessation has been reported to be sufficient in eliminating pigmentation [8]. However, in the present case, the patient claimed that she had stopped smoking one year previously and yet the gingival pigmentation had not disappeared. She asked us for a rapid way to eliminate this pigmentation.

The first procedure for the removal of gingival pigments, for aesthetical reasons, dates back to 1946, where an exfoliative method was used which was 90% phenol-based. The method presented limitations due to the quick draining and penetration in the gingival mucosa [16]. Since then several techniques have been developed including gingival abrasions (which involves the removal of the epithelium of pigmented areas using a high speed hand piece and diamond bur) [14, 26], gingivectomy (using a scalpel in which the gingival epithelium is removed as well as a layer of connective tissue, allowing secondary intention healing) [14, 17], cryosurgery (resorting to local necrosis through fast freezing), and gingival grafts [11, 13]. These techniques have been associated with some limitations such as lack of precision during surgery, lack of visibility of the tissue elimination especially for chemical and cryosurgery, hemorrhaging, and the need for dressings in gingivectomy techniques [11, 13, 17]. Lasers present several advantages in the treatment of oral tissues including precision cutting with possibility of histological analysis and a good visualisation of the surgical field in which there is coagulation during tissue elimination and therefore there is no necessity for any sutures [19–21]. When compared with conventional surgery, there are fewer risks of other complications commonly seen, such as pain, edema, and infection [19, 20, 31]. We could confirm these advantages in our case, as the patient did not report any pain, swelling, or other complications.

Several lasers have been used in depigmentation of oral melanotic macules [15, 24, 25, 28]. When looking at the different types of laser, it is possible to establish a relation between the type of biological effects and the wavelength used. The depth of penetration of each type of laser in the biological tissues varies according to the absorption of this energy [15, 24, 25, 28, 32]. Considering the localisation of the melanocytes in the epithelium, lasers with a superficial effect could be indicated, such as a CO_2_ laser. CO_2_ lasers are reported to cause minimal damage to the periosteum and bone beneath the gingiva but have a sufficient deep penetration (0.1 mm) to reach the melanocytes present pigmented areas [15, 33, 34]. Successful application of other lasers has been reported such as Nd:YAG or diode laser using melanin as chromophore [24, 25, 28]. However, the deep penetration of these wavelengths (4 to 6 mm) in the tissue could lead to several complications including bone necrosis or gingival fenestration as observed by Atsawasuwan et al. [24] when using Nd:YAG laser.

The final result we present in this case was achieved with one session. However, Nakamura et al. [22] reported the need for repeated sessions using a CO_2_ laser, in a continuous mode. The use of the pulsed mode as performed in the present case may avoid the carbonisation of tissues which allows a good visualisation of the surgical field. This could allow the elimination of the entire pigment present in the gingiva [33]. The number of cohort studies showing recurrence after laser appliance is limited. Ozbayrak et al. [15] observed no recurrence of gingival melanotic pigmentations in the 18 months after CO_2_ laser ablation. Kaya et al. [25] did not find any recurrence using Er:YAG and diode lasers in 20 patients with gingival melanotic pigmentations. However, repigmentation has been reported by others [17, 22]. Smoking may influence the success of the treatment. Esen et al. [33] performed depigmentation of gingival melanin pigmentation in 10 patients and observed a recurrence in 2 patients which were both smokers. Tobacco cessation may be mandatory to avoid repigmentation.

In conclusion, this case illustrates that CO_2_ lasers are a useful, effective, and safe application method in the removal of the gingival melanin pigmentation when tobacco cessation has not improved the appearance. However, longer cohort studies are needed for a better understanding of the potential benefits of this method.
